# Injection‑induced sciatic nerve injuries in Turkey: a public health and patient safety analysis of Supreme Court decisions

**DOI:** 10.1186/s12910-025-01283-5

**Published:** 2025-10-08

**Authors:** Elif Simin Issı, Furkan İncebacak

**Affiliations:** Department of Neurology, Faculty of Medicine, Afyonkarahisar Health Sciences University, Afyonkarahisar, Türkiye 03030 Turkey

**Keywords:** Sciatic nerve injury, Intramuscular injections, Medical malpractice, Patient safety, Judicial decisions, Public health policy

## Abstract

**Background:**

Intramuscular injections are routine interventions worldwide, yet when executed incorrectly they can cause sciatic nerve injury (SNI) that leaves patients with lifelong motor-sensory disability. Although international guidelines recommend the ventrogluteal site, the dorsogluteal region remains dominant in Turkey, potentially elevating risk. This study analysed Turkish Supreme Court decisions on injection-induced SNI from public-health, ethical and legal perspectives.

**Methods:**

We conducted a retrospective cross-sectional content analysis of all publicly available Supreme Court criminal and civil decisions issued between January 2006 and April 2025 that contained SNI-related keywords. After deduplication and relevance screening, 92 unique cases were eligible. Two investigators independently coded each judgment; disagreements were resolved by consensus. Variables recorded were legal category, injection site, clinical indication, drug class, injector profession, defendant identity, symptom latency, electrophysiological pattern, first-instance verdict and Supreme Court outcome. Frequencies and percentages were calculated with SPSS v.29.

**Results:**

Negligent bodily injury was the leading charge (50/92, 54%); 32% of files also sought monetary compensation. Gluteal injections accounted for 79% of cases, most administered for postoperative analgesia (33%) or antibiotic therapy (27%). Nurses performed 60% of injections, physicians 9%. Individual health professionals (physicians ± nurses) were defendants in 65% of lawsuits, while hospitals (alone or jointly) appeared in 23%. Symptoms emerged immediately or within 1 h in 76% of plaintiffs, and electromyography typically revealed severe axonal damage—predominantly of the peroneal division. The Supreme Court overturned 100% of firstinstance convictions and 32% of acquittals, most often citing inadequate expert evaluation (35%), contradictory reports (20%), uncertainty over negligence versus complication (18%) or missing informedconsent documentation (10%).

**Conclusions:**

Injection-related sciatic nerve injuries in Turkey remain potentially preventable. The entrenched use of the dorsogluteal site, limited anatomical awareness, inadequate informed consent practices, and inconsistencies in medico-legal evaluations continue to contribute to both patient harm and an increased burden of litigation. Transitioning to the ventrogluteal technique, mandating annual refresher training, standardising consent forms, and accrediting neurophysiology expert panels could help reduce both injury incidence and courtroom burden—advancing the WHO’s “zero harm” patient safety goal.

**Supplementary Information:**

The online version contains supplementary material available at 10.1186/s12910-025-01283-5.

## Introduction

Intramuscular (IM) injections are prevalent medical procedures; however, improper administration can result in significant complications. A notable complication is sciatic nerve injury, which may lead to enduring motor and sensory deficits, significantly impairing patients’ quality of life [1, 2]. Worldwide, approximately 16 billion injections are administered annually, a significant portion of which are superfluous or conducted under inadequate conditions [3]. Sciatic nerve injury can occur for a number of reasons, including direct mechanical trauma, the neurotoxic effects of certain medications, or ischaemic processes. The use of agents with significant neurotoxic potential, such as diclofenac sodium, has been shown to increase the risk of such injuries [4–6]. Multiple studies have shown that the injection site is a significant risk factor. Dorsogluteal injections carry an increased risk of nerve injury, while the ventrogluteal site is regarded as a safer option [7–9]. Reinforcing this evidence, a 2024 PRISMA-based Turkish systematic review encompassing 4,129 paediatric cases showed that ventrogluteal injections were associated with markedly fewer sciatic-nerve injuries and local reactions than either dorsogluteal or vastus lateralis sites [10]. In Turkey, the gluteal (specifically dorsogluteal) region continues to be predominantly taught and practiced as the intramuscular injection site in both clinical routines and nursing education programs [11–13]. Anatomical variations of the sciatic nerve substantially increase the risk of injury. Anatomical studies reveal that variations of the sciatic nerve occur in roughly 16–21% of the population [14, 15]. These variations can hinder healthcare professionals’ capacity to accurately administer injections, consequently elevating the risk of complications [16].

Despite the existence of World Health Organization (WHO) guidelines for safe injection practices, implementation of these guidelines remains poor due to a number of factors.

These include high workloads, lack of training and underutilization of critical incident reporting systems (CIRS) [17].

Research in Turkey suggests that injection-related sciatic nerve injury is a significant public health problem and is often the focus of medical malpractice litigation [18, 19]. Complementing these medicolegal data, a nationwide paediatric audit of injection-related sciatic neuropathy cases referred to the Council of Forensic Medicine demonstrated that nearly nine out of ten files were ultimately classified as ‘complications’ despite clear documentation gaps and high rates of dorsogluteal use [20]. A recent largescale national study by Vural and Erbaş (2024) analysed Supreme Court and Council of State compensation cases concerning informed consent using a comparable methodology and highlighted similar challenges in expert reporting and documentation [21]. Decisions of the Supreme Court (Yargıtay) in Turkey underscore the pivotal role of expert witness reports in distinguishing between complications and medical errors, while emphasising the legal ramifications of disregarding informed consent protocols [22, 23]. Under Turkish law, an injection-related sciatic-nerve injury (SNI) may give rise to concurrent criminal and civil proceedings: criminal liability is typically pursued under the negligent-injury and negligent-homicide provisions of Articles 85–89 of the Turkish Penal Code, while civil (tort- and contract-based) liability is anchored in Article 49 of the Turkish Code of Obligations—supplemented by Articles 502 and 506, which spell out the physician–patient mandate and its attendant duty of care—together with ancillary rules on employer responsibility and, in publicly run facilities, the administrative-law doctrine of “service fault.” By way of comparison, the landmark 2005 ruling of the Indian Supreme Court in *Jacob Mathew v. State of Punjab* introduced an “expert-opinion filter” designed to screen out unfounded criminal prosecutions of healthcare professionals, underscoring the importance of rigorous expert review in distinguishing unavoidable complications from negligent practice [24].

This study offers a novel contribution to the literature by: (i) presenting the largest Supreme Court (Yargıtay) dataset focused on injection-related sciatic nerve injury (SNI), (ii) simultaneously analyzing both criminal and civil legal dimensions, and (iii) quantitatively demonstrating deficiencies in medical documentation and the role of drug neurotoxicity.

## Methods

This national, retrospective, crosssectional content analysis examined all publicly available and anonymized Turkish Supreme Court decisions concerning injectioninduced sciatic nerve injuries published between January 1, 2006 and April 17, 2025 in the Turkish Supreme Court Decision Search System.

A comprehensive search was performed on 17 April 2025 using the Boolean combination of the keywords “injection”, “sciatic”, “nerve injury”, “malpractice”, “analgesic”, “muscle relaxant”, “antibiotic”, “gluteal”, and “hip”. The search retrieved 162 records; after removal of nonrelevant items (i.e., sciatic nerve injuries not related to intramuscular injection), 92 unique decisions were included in the analysis.

**Fig. 1:**
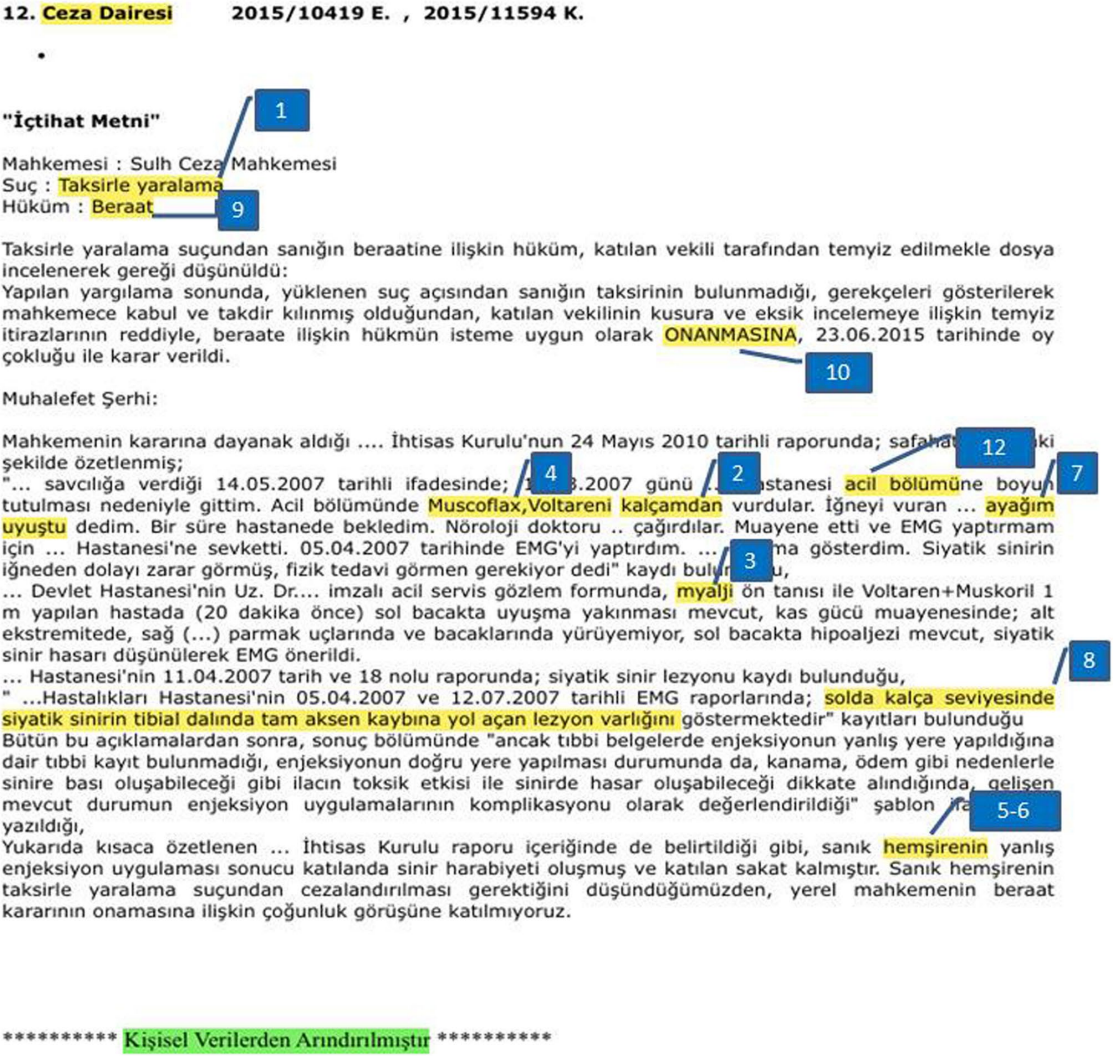
Coding framework and data elements extracted from Turkish Supreme Court decisions on injection-induced sciatic nerve injuries. (1) legal classification of the case; (2) injection site- *Although the judgment recorded the site simply as “gluteal*,*” it was coded as dorsogluteal in line with the anatomical location of the injury and prevailing clinical practice standards in Turkey*; (3) clinical indication; (4) class of drug administered; (5) professional category of the healthcare provider; (6) defendant profile; (7) time interval between injection and symptom onset; (8) electrophysiological findings; (9) verdict of the first-instance court; (10) Supreme Court decision; (11) grounds for reversal (where applicable); (12) hospital department where the injection was administered

From each fulltext decision, the research team prospectively extracted eleven variables: (1) legal classification of the case, (2) injection site (Although the injection site was recorded as “gluteal” in the court decision, it was coded as dorsogluteal based on the anatomical location of the complication and prevailing clinical practice standards in Turkey), (3) clinical indication, (4) class of drug administered, (5) professional category of the healthcare provider, (6) defendant profile, (7) time interval between injection and symptom onset, (8) electrophysiological findings, (9) verdict of the firstinstance court, (10) Supreme Court decision, and (11) grounds for reversal when applicable. Variable definitions and coding rules were finalized before data extraction and applied uniformly by two independent reviewers; disagreements were resolved by consensus. The coded variables are illustrated in an example judgment in Fig. 1.

The study was limited to descriptive statistics. Frequencies and percentages were calculated for all categorical variables, and results are reported in the text and summary tables. Because several subgroups contained fewer than five observations, no inferential statistical tests were performed.

All data analyzed were in the public domain and fully anonymized; therefore, ethical approval and informedconsent procedures were not required. No patients or members of the public were involved in the design or conduct of this study. Clinical trial number: not applicable.

## Results

In this study, 92 court cases related to alleged injection-induced sciatic nerve injury were analyzed, and both quantitative and qualitative findings were summarized. The tables present descriptive data on several key variables, including: the type of legal case (e.g., criminal negligence, compensation, medical malpractice), the anatomical site of injection (e.g., gluteal region, leg, groin), the clinical indication for injection (e.g., postoperative pain management, infection treatment), the type of medication administered (e.g., diclofenac, antibiotics), the professional background of the healthcare provider (e.g., nurse, physician, medical technician), the defendant profile (e.g., individual doctor, nurse, hospital institution), the onset time of symptoms, electrophysiological (EMG) findings, and the outcomes of both first-instance court rulings and Supreme Court decisions.

The data revealed that the majority of cases were classified as *negligent injury* lawsuits, with *compensation claims* also comprising a significant proportion (Table 1). It was noted that most injections were administered in the gluteal (hip) region; however, the laterality (right vs. left) was not consistently specified, and in 20.65% of the cases, the injection site was not indicated at all. Injections were most commonly performed for postoperative pain management and infection treatment purposes (Table 2). A considerable portion of the cases originated from emergency departments of public or private hospitals (77.17%), followed by inpatient wards after surgical procedures (15.22%).

**Table 1:** Frequency and proportional distribution of case types in litigation concerning injection-induced sciatic nerve injury (*n* = 92)

Case category	*n*	%
Negligent bodily injury	50	54.35
Compensation claim (any form of monetary damages)	29	31.52
Medical malpractice (injection neuropathy)	6	6.52
Misconduct in public office (acts of omission, etc.)	4	4.35
Determination of occupational accident	2	2.17
Determination of permanent work incapacity	1	1.09

**Table 2:** Primary clinical indications for injection in litigation concerning injection-induced sciatic nerve injury (*n* = 92)

Indication	*n*	%
Postoperative pain management	30	32.61
Infection treatment	25	27.17
Musculoskeletal/widespread pain	20	21.74
Headache/migraine	10	10.87
Not specified	4	4.35
Other	3	3.26

The administered drugs were predominantly diclofenac derivatives, although more than half of the case files did not specify the name of the medication used (Table 3). Nurses constituted the highest proportion (59.78%) among healthcare professionals administering the injections, which aligns with the fact that intramuscular injections are a routine part of nursing practice. Other practitioner groups included health officers/technicians (16.3%), physicians (8.7%), others (midwives, hospital staff, workplace health personnel, etc.) (8.7%), and in 6.52% of cases, the practitioner was not specified.

**Table 3:** Drug classes administered in litigation concerning injection-induced sciatic nerve injury (*n* = 92)

Drug class	*n*	%
Diclofenac group (e.g., Voltaren, Dicloron, Diclomec)	30	32.61
Antibiotics (e.g., Penadur, Ampisid, Sulcid, Pronopen)	6	6.52
Novalgin	4	4.35
Other (anaesthetic agents, combination preparations, agents other than Novalgin/Diclofenac)	3	3.26
Unspecified (“unknown”, “analgesic”, “name not provided”, etc.)	49	53.26

In most lawsuits, individual liability was attributed to either the physician or the nurse. However, a substantial number of cases also involved shared liability claims against both the hospital as a legal entity and the healthcare provider (Table 4). Clinical symptoms were reported to begin either almost simultaneously with the injection (76.09%) or within the first 24 h (5.43%). Electromyography (EMG) findings frequently indicated involvement of the peroneal branch, with a notable proportion of cases showing “complete (total)” (19.56%) or “severe (advanced) partial” (32.61%) axonal injury.

**Table 4:** Defendant parties in litigation concerning injection-induced sciatic nerve injury (*n* = 92)

Defendant category	*n*	%
Physician (individual)	30	32.61
Nurse/healthcare staff (individual)	20	21.74
Physician + Nurse (jointly sued)	10	10.87
Hospital (institutional legal entity)	15	16.30
Physician + Hospital (jointly sued)	6	6.52
Other (private hospital, public authority, infirmary, etc.)	5	5.43
Unspecified (not explicitly recorded)	6	6.52

A comparison between the decisions of first-instance courts and the rulings of the Court of Cassation revealed that approximately half of the acquittal verdicts were upheld, while more than one-third were overturned, and the remainder were approved with amendments. Notably, all convictions were reversed by the Court of Cassation. In civil lawsuits resulting in dismissal or partial acceptance, reversal was also frequent (Table 5), with the most common reasons being *“inadequate expert evaluation”* and *“contradictions between expert reports.”*

**Table 5:** Distribution of firstinstance and supreme court decisions in litigation concerning injection-induced sciatic nerve injury (*n* = 92)

Firstinstance verdict	Firstinstance *n* (%)	Supreme Court – Affirmed *n* (%)	Supreme Court – Reversed *n* (%)	Supreme Court – Affirmed with modification *n* (%)	Supreme Court – Other/Lack of jurisdiction *n* (%)
Acquittal	41 (44.57)	19 (46.3)	13 (31.7)	6 (14.6)	3 (7.3)
Conviction	10 (10.87)	0 (0.0)	10 (100.0)	0 (0.0)	0 (0.0)
Dismissed	20 (21.74)	10 (50.0)	9 (45.0)	0 (0.0)	1 (5.0)
Partially granted	15 (16.30)	3 (20.0)	10 (66.7)	1 (6.7)	1 (6.7)
Lack of jurisdiction/Other	6 (6.52)	3 (50.0)	1 (16.7)	0 (0.0)	2 (33.3)

In cases involving pediatric patients (8 files), deficiencies in evaluating injection technique, anatomical considerations, and the informed consent process—particularly parental notification—were commonly cited as grounds for reversal due to “insufficient examination.” A significant number of case files lacked a documented informed consent form, or included incomplete information, which contributed to challenges in distinguishing between medical negligence and complications during legal proceedings. Disability percentages were specified in only 6 cases (6.52%), indicating that the extent of permanent impairment was often not clearly documented.

Of the 29 civil-compensation suits in our dataset, 11 Supreme Court (Yargıtay) decisions disclose an explicit payout figure. Five of those 11 judgments involve first-instance courts that actually awarded damages: 176 408 TRY in the single nurse-liability case (the only award that has since been affirmed on appeal); 113 066 TRY, 30 481 TRY and 3 000 TRY in three separate hospital-liability cases; and 20 000 TRY in a joint physician–hospital matter. The five amounts span 3 000–176 408 TRY, yielding a mean of 68 591 TRY and a median of 30 481 TRY. Averaged by defendant type, hospitals awarded ≈ 48 849 TRY (*n* = 3), the nurse case 176 408 TRY, and the physician–hospital case 20 000 TRY.

Claim-versus-award figures were available in seven files: courts reduced patient demands by a mean of ≈ 44 000 TRY (median ≈ 52 000 TRY), with the steepest proportional reduction (~ 80%) in the physician–hospital case. Because only one award is legally final, the sample is small and heterogeneous, and the rulings span 2006–2025—a period of pronounced inflation and a 2005 currency redenomination—these numbers are presented descriptively without inferential statistics; their real purchasing power varies substantially across the study interval.

## Discussion

In this study, 92 court cases related to sciatic nerve injuries following intramuscular injection were analysed. The findings were evaluated in light of the existing literature and from both legal and public health perspectives. Our results highlight not only individual clinical errors but also structural deficiencies within the healthcare system and gaps in patient safety protocols. These observations suggest that national standardisation of intramuscular injection practice and more robust incidentreporting systems could help countries progress toward the World Health Organization’s Global Patient Safety Action Plan 2021–2030 target of eliminating avoidable harm in health care [25].

The determination of liability or complication in postinjection sciatic nerve injury cases appears to be influenced by the thoroughness of expert reports, anatomical accuracy, informedconsent quality, and completeness of medical documentation. The reversal of all criminal convictions by the Court of Cassation indicates that establishing fault in criminal courts may require stringent evidentiary and technical standards. Cases involving nurses, physicians, or hospitals in various combinations underline the importance of teambased responsibility and institutional protocols. The reasons underlying the more frequent attribution of liability to nurses could not be statistically evaluated, as the detailed data required to assess potential contributing factors were absent in the majority of case files. Enhancing standardisation, risk management, and comprehensive recordkeeping in intramuscular injection practices could therefore help reduce both the number of legal disputes and the ambiguity surrounding accountability.

Within the international literature, injectionrelated nerve injury is often discussed in terms of ‘preventable medical error’ or ‘professional negligence’ [7, 8]. As Studdert et al. (2006) note, even in the absence of demonstrable malpractice, litigation can still result in compensation. These observations highlight the potential importance of evaluating factors such as patient safety, the drug administered, anatomical site selection, and informedconsent processes [26]. This perspective also illustrates how scientific evidence can inform legal and ethical frameworks aimed at improving health protection and service quality [8, 27].

In the present study, 54.35% of the cases were criminal prosecutions for negligent injury and 31.52% were civil compensation claims. These proportions indicate that sciaticnerve injuries after injections fairly often reach the courts and may entail serious consequences for providers and institutions. When compensation was awarded (5/11 cases), sums ranged from 3 000 TRY to 176 408 TRY (mean ≈ 68 591; median ≈ 30 481). On average, hospitals paid ≈ 48 849 TRY (*n* = 3), the single nurse-liability case 176 408 TRY, and the joint physician–hospital case 20 000 TRY. In seven files with both claim and award, courts trimmed claims by **~** 44 000 TRY on average (median ≈ 52 000). These descriptive figures span 2006–2025 and are not inflation-adjusted or statistically tested. Analysis of Court of Cassation rulings showed that, although some lowercourt decisions cited “technical error”, many were later overturned (e.g., all 10 criminal convictions and 39 of 71 civil judgments), suggesting that higher courts apply stringent evidentiary standards before confirming liability. Although the level of evidence is limited, this observation is supported by a reversal decision issued by the 12th Criminal Chamber of the Turkish Supreme Court (Case No: 2014/20069 E., 2015/11137 K.), in which the expert report was deemed to lack adequate scientific justification in distinguishing between technical error and complication. These reversal rates are broadly consistent with those reported by Vural and Erbaş in their 2024 national review of 126 informedconsent malpractice cases, which also highlighted documentation gaps and expertreport inconsistencies [21].

This discrepancy may reflect differences in perspective between Turkish judicial practice and international approaches, particularly in how medical error is distinguished from unavoidable complication. Intramuscular injections into the dorsogluteal region are widely reported to carry a higher risk of sciatic nerve injury [7, 8], whereas the ventrogluteal site is generally considered safer because of its anatomical separation from major nerves and vessels [27]. Anatomical variations between the piriformis muscle and the sciatic nerve—described in ~ 11–21% of individuals—could further increase the likelihood of traumatic injury. These considerations underscore the need for clinicians to assess anatomical risk before injection and choose the safest site available. This requirement is illustrated by the decision of the 12th Criminal Chamber (Case No. 2014/22119 E., 2015/16181 K.), in which the sciatic nerve injury that developed after a gluteal injection in a 6-year-old pediatric case was deemed an “unforeseeable complication,” and the acquittal was upheld; however, the dissenting opinion considered the nurse’s failure to perform anatomical landmarking as a fault.

In our series, gluteal placement was documented in 79.35% of cases. Symptoms developed within an hour in 76.09% of patients, and EMG confirmed sciatic involvement in most (52.18% with total or neartotal damage and 17.39% with predominant peronealbranch involvement). Taken together, these observations are consistent with the view that many such injuries are potentially preventable. Although current guidelines describe “safeinjection” measures—such as hand hygiene and refraining from needle recapping [28]—breaches have been reported in busy settings like emergency departments and operating theatres [17]. Many of our cases appear to illustrate these anatomically and procedurally related risks.

In fastpaced units (e.g., emergency rooms, outpatient clinics), avoidable complications may arise when anatomical landmarks are not marked adequately, the needle length or angle is unsuitable, or staff training is insufficient [5, 9]. While such “active errors” are often captured by Critical Incident Reporting Systems (CIRS), corrective actions are reportedly implemented inconsistently [17]. Consequently, despite existing regulations and guidelines, implementation gaps are likely to persist in daily practice. The fact that 77.17% of events in our sample occurred in emergency or postoperative units suggests that high patient turnover, time pressure and limited staffing may hamper optimal care. Rather than focusing solely on individual blame, these findings point to the potential value of systemlevel policies aimed at strengthening standardisation and safety in intramuscularinjection practice.

Postoperative pain management (32.61%) and infection treatment (27.17%) were the most common indications for intramuscular injections. These figures suggest that IM injections remain a widely used option for rapid symptom relief or supportive therapy; however, our data do not show consistent documentation of a formal risk–benefit assessment. In many legal files, contributing factors included inadequate training, incomplete informedconsent forms, and limited awareness of anatomical risks.

The frequent use of potentially neurotoxic agents such as diclofenac (32.61% of cases where the drug was specified) and the absence of medication details in 53.26% of records point to notable documentation gaps. Such deficiencies may increase patientsafety and medicolegal risks [18]. Overall, the findings highlight the importance of robust medicationmanagement protocols and comprehensive healthinformation systems.

Additionally, although nurses administered most intramuscular injections, several studies have identified gaps between their theoretical knowledge and everyday practice [29, 30]. The landmark report *To Err Is Human* [31] showed that many preventable medical errors are linked to systemdesign shortcomings, and it highlighted the value of institutional policies, standardised protocols, and effective monitoring.

In our dataset, 59.78% of injections were delivered by nurses. Some court files focused mainly on individual negligence, with limited discussion of organisational accountability or hospital management’s responsibilities. Such an emphasis on personal fault may obscure wider system weaknesses. Strengthening institutional policies and regulatory oversight—both within hospitals and at the Ministry of Health level—could therefore support sustainable improvements in intramuscularinjection safety.

The prolonged duration of legal proceedings and the occurrence of permanent sequelae from preventable, hospitalacquired injuries underscore the pressing need for multilayered policies aimed at improving patient safety. In this context, placing systematic emphasis on the ventrogluteal injection technique in nursing and medical curricula, ensuring complete implementation of informedconsent and documentation processes, activating reporting tools such as Critical Incident Reporting Systems (CIRS), and aligning expertwitness standards with international benchmarks could make an important contribution to safer practice. It would be beneficial for the Ministry of Health and professional bodies to act as key partners in formulating national guidelines and developing effective riskmanagement strategies in hospitals. Adopting these measures may help minimise errors that appear as preventable complications, thereby improving patient safety and reducing the financial and psychological burden associated with lengthy litigation.

In the international literature, intramuscular injections administered to the dorsogluteal region are often discussed as “preventable medical errors,” with responsibility extending beyond individual negligence to systemic shortcomings [7, 8]. Within the patientsafety framework, systemlevel measures—such as education, standardised guidelines and regulatory oversight—are recommended, particularly for routine procedures like injections [17]. Nevertheless, as in many countries, the “complication” defence in Turkey relies heavily on expertwitness reports; inconsistencies or methodological weaknesses in those reports may influence legal outcomes.

Our review of 92 Supreme Court (Court of Cassation) rulings showed that around 55**%** of firstinstance decisions were overturned. In all ten criminal convictions, the Supreme Court reversed the judgment. Among acquittals, 46.3% were upheld, while the remainder were reversed or modified. In civil cases, 45**%** of dismissal rulings and 66.7**%** of partialacceptance judgments were not upheld.

When reversal reasons were grouped, the most common was “inadequate expert evaluation or incomplete investigation” (35%), followed by “conflicting expert reports” (20%), For instance, in its decision numbered 2018/1759 E., 2019/208 K., the 21 st Civil Chamber reversed the judgment on the grounds that the initial examination was deemed insufficient. “Uncertainty in distinguishing between complication and negligence” (18%) and “lack of informed consent” (10%). Technical and procedural errors were also notable factors.

These findings link patientsafety gaps in intramuscularinjection practice not only to individual clinical errors but also to structural shortcomings—such as limited standardised training, weak incidentreporting systems, and insufficient anatomicalrisk assessment—and indicate that these issues should be addressed through coherent national policies and multistakeholder collaboration. The World Health Organization’s Global Patient Safety Action Plan 2021–2030 highlights standardisation of safe clinical processes, multidisciplinary training, and institutional accountability as practical pathways that could help reduce preventable nerve injuries associated with intramuscular injections. Taken together, our results offer additional evidence that may support adoption of WHO’s multisectoral “zero harm” approach at the national level.

### Strengths and limitations of the study

Using Supreme Court rulings as the primary data source provides a valuable national perspective. Nevertheless, the retrospective design and missing data in some judgments (e.g., unspecified drug names) limit the generalisability of our findings. The absence of complete covariate information prevented multivariate analyses and thus curtailed a deeper exploration of clinicallegal relationships. Our ability to conduct comprehensive nurse versusdoctor liability modelling, professionspecific compensation comparisons, and claim–award gap analysis was limited by incomplete information in publicly released judgments. Future studies that obtain fulltext court files or linked hospital records could address this limitation. In addition, securing multicentre prospective incident reports would enable more nuanced modelling of risk factors.

## Conclusion

This study suggests that although sciatic nerve injuries following intramuscular injections are often labeled as “complications,” they may, in many instances, be preventable. The predominance of dorsogluteal injections, the absence of standardized education and clinical protocols, deficiencies in informed consent procedures, and the limited effectiveness of institutional safety reporting systems (such as CIRS) all point to systemic vulnerabilities in healthcare delivery. Inconsistencies in expert witness reports and the frequent reversals observed in judicial rulings illustrate the misalignment between clinical realities and legal assessments.

From a public health perspective, reducing such potentially preventable neurological injuries is unlikely to be achieved solely by assigning individual responsibility; therefore, broader structural reforms may be needed. These could include integrating safer injection techniques (e.g., the ventrogluteal approach) into training curricula, standardising consent and documentation processes, improving the quality of expert reports, and reinforcing institutional accountability through national health policy measures. Such a holistic approach could foster progress in patient safety, institutional learning and judicial consistency, while providing a framework for solutions that are ethically, legally and qualitatively aligned with contemporary public health principles. 

## Supplementary Information

Supplementary Material 1.

## Data Availability

The full-text judgments used in this study are freely accessible via the Turkish Court of Cassation database (https://karar.yargitay.gov.tr). The coded extraction spreadsheet can be obtained from the corresponding author on reasonable request.
