# Supplementary material for: Injection‑induced sciatic nerve injuries in Turkey: a public health and patient safety analysis of Supreme Court decisions
Source: BMC Med Ethics. 2025 Oct 8;26:130. doi: 10.1186/s12910-025-01283-5 (PMC12509378; doi:10.1186/s12910-025-01283-5)
Supplement: Supplementary file 1 — Supplementary Material 1. [file 12910_2025_1283_MOESM1_ESM.doc]

STROBE Statement—Checklist of items that should be included in reports of ***cross-sectional studies***

|  | Item No | Recommendation |
| --- | --- | --- |
| **Title and abstract** | 1 | (*a*) Indicate the study’s design with a commonly used term in the title or the abstract🡪 **Yes – Title (l. 1‑2) states “Analysis of Supreme Court Decisions” and Abstract l. 10‑11 says “retrospective cross‑sectional content analysis”.** |
|  |  | 1. (*b*) Provide in the abstract an informative and balanced summary of what was done and what was found🡪 **Yes – Fully addressed in Abstract (lines 4–28)** |
| Introduction | | |
| Background/rationale | 2 | Explain the scientific background and rationale for the investigation being reported🡪 **Yes – Fully Intro l. 41‑66; highlights public‑health burden & medico‑legal gap.** |
| Objectives | 3 | State specific objectives, including any prespecified hypotheses🡪**Yes – Abstract, line 9, Introduction, lines 62–66.** |
| Methods | | |
| Study design | 4 | Present key elements of study design early in the paper🡪 **Yes – Mentioned in Abstract (lines 10–11) and Methods (lines 68–71)** |
| Setting | 5 | Describe the setting, locations, and relevant dates, including periods of recruitment, exposure, follow-up, and data collection🡪 **Yes – Turkish Supreme Court, 1 Jan 2006 to 17 Apr 2025; search conducted on 17 Apr 2025 (lines 68–73).** |
| Participants | 6 | (*a*) Give the eligibility criteria, and the sources and methods of selection of participants🡪 **Yes – Described in Methods (lines 72–87)** |
| Variables | 7 | Clearly define all outcomes, exposures, predictors, potential confounders, and effect modifiers. Give diagnostic criteria, if applicable🡪 **Yes – Eleven variables defined (lines 76–82)** |
| Data sources/ measurement | 8* | For each variable of interest, give sources of data and details of methods of assessment (measurement). Describe comparability of assessment methods if there is more than one group🡪 **Yes – Same lines; coded independently by 2 reviewers, consensus process described l. 82‑84.** |
| Bias | 9 | Describe any efforts to address potential sources of bias🡪 **Yes – Double-reviewer consensus approach (lines 82–84)** |
| Study size | 10 | Explain how the study size was arrived at🡪 **Yes –Entire universe of eligible cases within period; state explicitly in Methods l. 68‑73, 162 screened, 92 included (lines 73–76)** |
| Quantitative variables | 11 | Explain how quantitative variables were handled in the analyses. If applicable, describe which groupings were chosen and why🡪 **Yes – Frequencies and percentages used; no continuous variable groupings (lines 87–95, Tables 1–5)** |
| Statistical methods | 12 | (*a*) Describe all statistical methods, including those used to control for confounding🡪**Yes – Methods p. 3 (L94-97)** |
|  |  | (*b*) Describe any methods used to examine subgroups and interactions🡪**Not applicable** |
|  |  | (*c*) Explain how missing data were addressed🡪**Yes – Methods p. 3 (L95-96)** |
|  |  | (*d*) If applicable, describe analytical methods taking account of sampling strategy🡪**Not applicable** |
|  |  | (*e*) Describe any sensitivity analyses🡪**Not applicable** |
| Results | | |
| Participants | 13* | (a) Report numbers of individuals at each stage of study—eg numbers potentially eligible, examined for eligibility, confirmed eligible, included in the study, completing follow-up, and analysed🡪**Yes – Methods (L 72-76)** |
|  |  | (b) Give reasons for non-participation at each stage🡪**Yes – p. 4 (L 72-76)** |
|  |  | (c) Consider use of a flow diagram🡪**Given the simple two-step selection (162 ➜ 92), a PRISMA-style flow diagram was deemed optional; numeric counts are fully reported in the text.** |
| Descriptive data | 14* | (a) Give characteristics of study participants (eg demographic, clinical, social) and information on exposures and potential confounders🡪**Yes – Results L.102-147 (Tables 1–5)** |
|  |  | (b) Indicate number of participants with missing data for each variable of interest🡪**Yes – Results Add footnotes in each table indicating ‘unspecified/unknown’ counts (e.g., injection site unspecified = 40 cases 43.48 %).** |
| Outcome data | 15* | Report numbers of outcome events or summary measures🡪**Yes – Results (Table 5)** |
| Main results | 16 | (*a*) Give unadjusted estimates and, if applicable, confounder-adjusted estimates and their precision (eg, 95% confidence interval). Make clear which confounders were adjusted for and why they were included🡪**Not applicable – Results are purely descriptive.** |
|  |  | (*b*) Report category boundaries when continuous variables were categorized🡪Continuous variables were not analysed. |
|  |  | (*c*) If relevant, consider translating estimates of relative risk into absolute risk for a meaningful time period🡪**Not applicable:** no risk estimates were calculated; study is a retrospective descriptive content analysis of court decisions. |
| Other analyses | 17 | Report other analyses done—eg analyses of subgroups and interactions, and sensitivity analyses🡪**Not applicable** |
| Discussion | | |
| Key results | 18 | Summarise key results with reference to study objectives🡪**Yes – Discussion (L150-157)** |
| Limitations | 19 | Discuss limitations of the study, taking into account sources of potential bias or imprecision. Discuss both direction and magnitude of any potential bias🡪**Discussion (L274-280)** |
| Interpretation | 20 | Give a cautious overall interpretation of results considering objectives, limitations, multiplicity of analyses, results from similar studies, and other relevant evidence🡪**Yes – Discussion & Conclusions:** **Discussion l. 149‑203 synthesises key findings with objectives and prior literature; lines 227‑242 and 251‑264 weigh systemic implications; Conclusion l. 283‑297 reiterates public‑health significance while noting study limitations** |
| Generalisability | 21 | Discuss the generalisability (external validity) of the study results🡪**Discussion L306-309:** Results are based on Turkish Supreme-Court rulings; therefore findings may not be directly generalisable to other legal systems or healthcare contexts. Retrospective design and missing clinical detail further limit external validity, so conclusions should be applied with caution outside Turkey. |
| Other information | | |
| Funding | 22 | Give the source of funding and the role of the funders for the present study and, if applicable, for the original study on which the present article is based🡪 **Yes – Funding section L317:** *“This research received* ***no external funding****. The article-processing charge will be paid by the authors.”* Because there was **no external funder**, there was **no funder role** in study design, data collection, analysis, interpretation, or manuscript preparation; and there is **no prior (“original”) study** on which the present article is based. |

*Give information separately for exposed and unexposed groups.
